# An immunohistochemical study of the antinociceptive effect of calcitonin in ovariectomized rats

**DOI:** 10.1186/1471-2474-9-164

**Published:** 2008-12-15

**Authors:** Bunji Takayama, Shin-ichi Kikuchi, Shin-ichi Konno, Miho Sekiguchi

**Affiliations:** 1 Department of Orthopedic Surgery, Fukushima Medical University School of Medicine, 1-Hikarigaoka, Fukushima City, Fukushima, 960-1295, Japan

## Abstract

**Background:**

Calcitonin is used as a treatment to reduce the blood calcium concentration in hypercalcemia and to improve bone mass in osteoporosis. An analgesic effect of calcitonin has been observed and reported in clinical situations. Ovariectomaized (OVX) rats exhibit the same hormonal changes as observed in humans with osteoporosis and are an animal model of postmenopousal osteoporosis. The aim of this study to investigate antinociceptive effect of calcitonin in OVX rats using the immunohistochemical study.

**Methods:**

We assessed the antinociceptive effects of calcitonin in an ovariectomized (OVX) rat model, which exhibit osteoporosis and hyperalgesia, using the immunohistochemical method. Fifteen rats were ovariectomized bilaterally, and ten rats were received the same surgery expected for ovariectomy as a sham model. We used five groups: the OVX-CT (n = 5), the sham-CT (n = 5), and the OVX-CT-pcpa (n = 5) groups recieved calcitonin (CT: 4 U/kg/day), while OVX-vehi (n = 5) and the sham-vehi (n = 5) groups received vehicle subcutaneously 5 times a week for 4 weeks. The OVX-CT-pcpa-group was given traperitoneal injection of p-chlorophenylalanine (pcpa; an inhibitor of serotonin biosynthesis) (100 mg/kg/day) in the last 3 days of calcitonon injection. Two hours after 5% formalin (0.05 ml) subcutaneously into the hind paw, the L5 spinal cord were removed and the number of Fos-immunoreactive (ir) neurons were evaluated using the Mann-Whitney-U test.

**Results:**

The numbers of Fos-ir neurons in the OVX-CT and sham-CT groups were significantly less than in the OVX-vehi and sham-vehi groups, respectively (p = 0.0090, p = 0.0090). The number of Fos-ir neurons in the OVX-CT-pcpa-group was significantly more than that of the OVX-CT-group (p = 0.0283), which means pcpa inhibits calcitonin induced reduction of c-Fos production.

**Conclusion:**

The results in this study demonstrated that 1) the increase of c-Fos might be related to hyperalgesia in OVX-rats. 2) Calcitonin has an antinociceptive effect in both OVX and sham rats. 3) The central serotonergic system is involved in the antinociceptive properties of calcitonin.

## Background

Calcitonin is a polypeptide hormone that is secreted into the general circulation by the parafollicular cells of the mammalian thyroid gland, and it regulates the blood calcium concentration and bone metabolism by acting on osteoclasts. Calcitonin is used as a treatment to reduce the blood calcium concentration in hypercalcemia and to improve bone mass in osteoporosis. An analgesic effect of calcitonin has been observed and reported in clinical situations, and in randomized controlled studies calcitonin has been found to produce an analgesic effect in patients with osteoporotic vertebral compression fractures [1,2], reflex sympathetic dystrophy [3], and cancer pain [4]. Menopause is well known to be one of the essential causes of osteoporosis in human [5], and since the most important change following menopause is the depression of estrogen, which regulate expression of various genes [6], depletion of estrogen decreases the amounts of gene products, including receptors and peptides, required for modulation of nociceptive transmission. Ovariectomaized (OVX) rats exhibit the same hormonal changes as observed in humans with osteoporosis and are an animal model of postmenopausal osteoporosis. OVX rats have been used to evaluate the therapeutic effect of analgesic drugs [7]. A significant reduction in the latencies of tail withdrawal from hot water [8] and long-term formalin-induced licking [9] has also been reported in OVX rats, and because of this OVX is thought to induce hyperalgesia in rats.

The c-fos proto-oncogene is one of the immediate-early genes, and its expression by both noxious and non-noxious stimuli [10]. It encodes a nuclear phosphoprotein, c-Fos, that acts as a transcription factor by binding to DNA or DNA-binding proteins in the promoter region. C-Fos is expressed in postsynaptic neurons of the dorsal horn of the spinal cord at an early stage of nociception, and temporarily in response to various stimuli from primary afferent neurons, and its level of expression depends on the types and strength of the stimuli [11-14]. C-Fos expression is inhibited by applications of morphine and non-steroidal anti-inflammatory drugs (NSAIDs) [15-18]. Because of these facts, the c-Fos expression in spinal cord neurons has been used as a functional marker of nociception in many studies [11,15-19]. The aim of the present study was to assess the hyperalgesia in OVX rats and the antinociceptive effect of calcitonin in OVX rats by measuring the c-Fos expression as a marker of nociception.

## Methods

Twenty-five female Sprague-Dawley rats (200–250 g) were used. The experiment was performed with the approval of the Animal Research Committee of the Fukushima Medical University, and the animals were cared for in the Experimental Animal Center of Fukushima Medical University.

**Figure 1:**
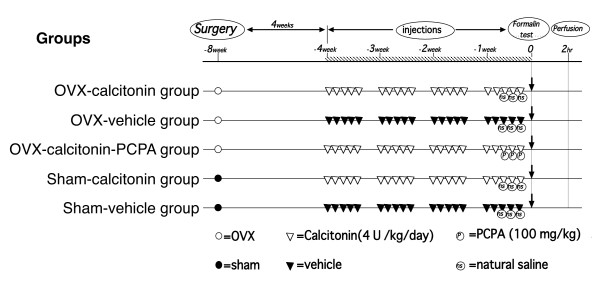
**Ovariectomy or a sham operation was performed in each group, and from postoperative week 4 onward calcitonin or vehicle was injected 5 times a week for 4 weeks**. PCPA was injected on the final 3 days of calcitonin injection. A formalin test was conducted in postoperative week 8, and two hours later, the animals in each group were perfused, and tissue was removed.

The rats were anesthetized by inhalation of ethyl ether (99%, Diethyl Ether, Wako Pure Chemical Industries, Ltd., Osaka, Japan). Bilateral ovariectomy was performed according to the method reported by Shibata K et al [20] to create an OVX model. Rats whose ovaries were exposed but not excised were used as a sham model. The operations and drug administrations were performed according to the schedule shown in the Figure 1. Four weeks after surgery, a synthetic derivate of eel calcitonin, ([Asu^1'7^]eel calcitonin) (4 U/kg/day) (Elcatonin, Asahi Kasei Co., Tokyo, Japan) was subcutaneously injected into the back of rats 5 times a week for 4 weeks. The vehicle was injected as a control. P-chlorophenylalanine (PCPA; Sigma, MO, USA), 100 mg/kg/day, an inhibitor of serotonin biosynthesis, was intraperitoneally injected on the final 3 days of calcitonin administration. Normal saline was injected as a control. The following 5 groups were established: a) a group repeatedly subcutaneously injected with calcitonin after OVX (OVX-calcitonin group, n = 5), b) a group repeatedly subcutaneously injected with vehicle after OVX (OVX-vehicle group, n = 5), c) a group intraperitoneally injected with PCPA after OVX in addition to being repeatedly subcutaneously injected with of calcitonin (OVX-calcitonin-PCPA group, n = 5), d) a group repeatedly subcutaneously injected with calcitonin after sham operation (sham-calcitonin group, n = 5), and e) a group repeatedly subcutaneously injected with vehicle after sham operation (sham-vehicle group, n = 5). After surgery and injections, the formalin test was performed as an acute noxious stimulus with subcutaneously injected with 5% formalin (0.050 ml) (Wako Pure Chemical Industries, Ltd. Osaka, Japan) into the left hind paw. All rats were perfused two hours after formalin test.

### C-Fos immunohistochemistry

The samples of spinal cord were prepared and immunohistochemically stained by methods based on those described by Hamba et al [19]. Two hours after the formalin test, the rats were deeply anesthetized with ethyl ether and they intracardially perfused with 200 ml of a 4% paraformaldehyde-0.1 M phosphate buffer (PBS) solution. After perfusion, the L5 segment of spinal cord was excised. The spinal cord was immersed in the 4% paraformaldehyde-0.1 M PBS solution for one hour, then in a 10% sucrose-PBS solution for 24 hours, and finally in a 20% sucrose-PBS solution for 24 hours. A 40-μm frozen section was then cut with a microtome. It was collected as a floating section in 0.1 M PBS, and it was immersed in 0.1 M PBS containing 0.2%Triton X for three days. All sections were immunostained for c-Fos protein by the avidin-biotin peroxidase (ABC) technique with commercial ABC kits (Vector Labs, Burlingame, CA). To eliminate confusion between small immunoreactive neurons and red blood cells, the protocol included hydrogen peroxide pretreatment. The sections were incubated in a solution of normal goat serum and then for 48 hours at 4°C in primary antiserum, which was a rabbit polyclonal antibody directed against a synthetic peptide that corresponded to the N-terminal of the c-Fos protein (Santa Cruz biotechnology, Inc., CA, USA) at a dilution of 1:3000. The reaction was visualized with 0.006% hydrogen peroxide and 0.02% diaminobenzidine (DAB, Wako Pure Chemical Industries, Ltd. Osaka, Japan). Tissue sections were thoroughly rinsed with Tris buffer, mounted onto MAS-coated slides (Matsunami Grass Industries, Ltd. Osaka, Japan), dried, dehydrated in a graded alcohol series, cleared in xylene and coverslipped.

### Quantitation of c-Fos immunoreactive neurons

All sections were digitized with a CCD camera (Axio Cam Mrc, Carl Zeiss, Hallbergmoos, Germany) equipped with a computer-assisted image analysis system (KS 100 imaging system, Carl Zeiss, Hallbergmoos, Germany). All data analysis procedures were performed blindly with respect to the experimental condition of the animal. The five sections from each rat that containing highest numbers of c-Fos immunoreactive neurons were selected for analysis. The spinal cord was divided into 3 regions [21]: (1) laminae I–II (the superficial laminae); (2) laminae III–IV (the nucleus proprius); (3) laminae V–VI (the neck of the dorsal horn), and the average number of c-Fos immunoreactive neurons in each region was calculated by averaging the counts made in the five sections from each rat.

### Statistical analysis

Comparisons between the numbers of c-Fos immunoreactive neurons in two groups were made by the Mann-Whitney's U test. A p-value less than 0.05 was considered indicative of a statistically significant difference.

## Results

### c-Fos expression in laminae I–II of the dorsal horn of the spinal cord

**Figure 2:**
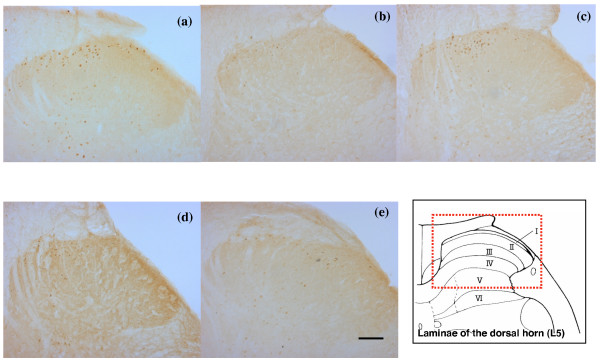
**Photomicrographs of transverse sections of L5 level of the lumbar spinal cord illustrating the distribution of c-Fos immunoreactive neurons in the dorsal horn in the OVX-vehicle group (a), OVX-calcitonin group (b), OVX-calcitonin-PCPA group (c), sham-vehicle group (d), and sham-calcitonin group (e)**. Scale bar, 100 μm. The Figure shows a section of dorsal horn at the L5 level [21].

**Figure 3:**
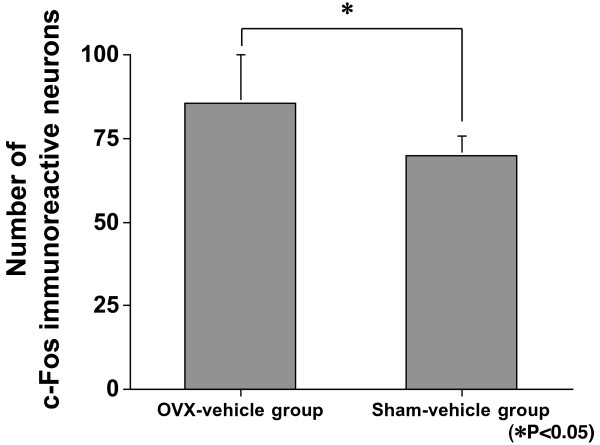
**Expression in laminae I–II: OVX-vehicle group vs sham-vehicle group**. The number of c-Fos immunoreactive neurons in the OVX-vehicle group was significantly greater than in the sham-vehicle group (p = 0.0367).

**Figure 4:**
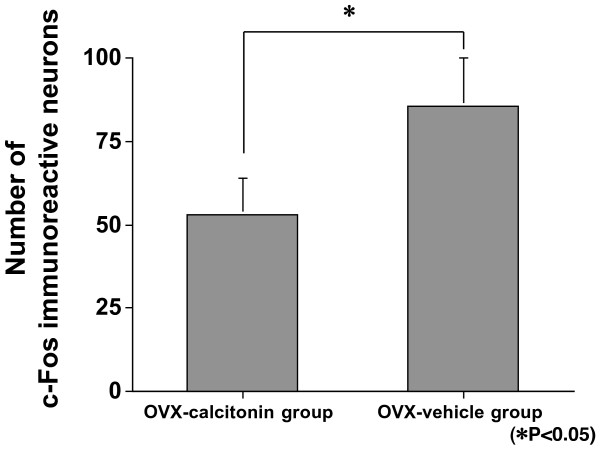
**Expression in laminae I–II: OVX-calcitonin group vs OVX-vehicle group**. There were significantly fewer c-Fos immunoreactive neurons in the OVX-calcitonin group than in the OVX-vehicle group (p = 0.0090).

**Figure 5:**
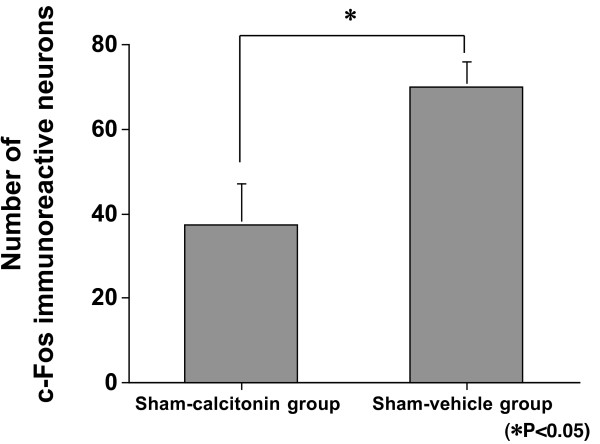
**Expression in laminae I–II: Sham-calcitonin group vs sham-vehicle group**. There were significantly fewer c-Fos immunoreactive neurons in the sham-calcitonin group than in the sham-vehicle group (p = 0.0090).

**Figure 6:**
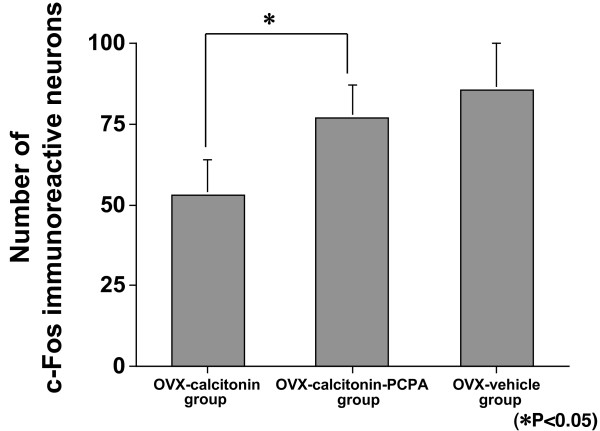
**Expression in laminae I–II: OVX-calcitonin-pcpa group vs OVX-calcitonin group and OVX-vehicle group**. There were significantly more c-Fos immunoreactive neurons in the OVX-calcitonin-PCPA group than in the OVX-calcitonin group (p = 0.0283), but the difference from the OVX-vehicle group was not significant (p = 0.4034).

Figure 2 shows illustrative cases of c-Fos immunoreactive neurons in each of the groups. The number of c-Fos immunoreactive neurons in the OVX-vehicle group was significantly greater than in the sham-vehicle group (p = 0.0367; Figure 3), indicating that OVX in rats result in a greater number of neurons in dorsal horn of the spinal cord that produce c-Fos after formalin test. There were significantly fewer c-Fos immunoreactive neurons in the OVX-calcitonin group than in the OVX-vehicle group (p = 0.0090; Figure 4), and significantly fewer c-Fos immunoreactive neurons in the sham-calcitonin group than in the sham-vehicle group (p = 0.0090; Figure 5). These findings mean that repeated calcitonin injection of both the OVX rats and the sham-operated rats result in fewer neurons in the dorsal horn of spinal cord that produced c-Fos after the formalin test. There were significantly more c-Fos immunoreactive neurons in the OVX-calcitonin-PCPA group than in the OVX-calcitonin group (p = 0.0283; Figure 6), but the difference from the OVX-vehicle group was not significant (Figure 6), indicating that the effect of repeated doses of calcitonin that decreases the number of c-Fos-producing neurons after the formalin test was abolished when the serotonin synthesis inhibitor was administered.

### c-Fos expression in laminae III–IV and laminae V–VI

**Figure 7:**
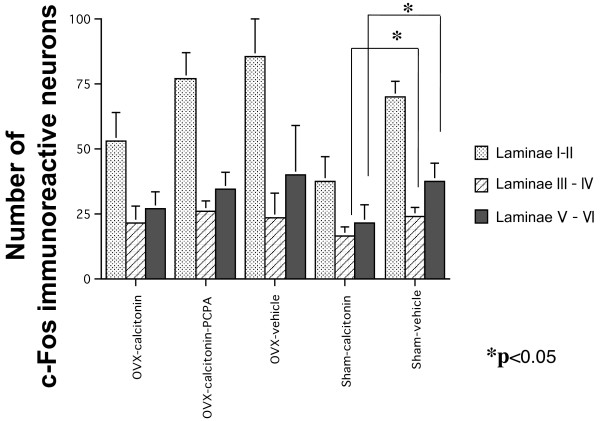
**c-Fos expression in laminae III–IV and laminae V–VI of the dorsal horn**. The results of comparison of the number of c-Fos immunoreactive neurons between groups in laminae III–IV and laminae V–VI were similar to the results in laminae I–II, however the only significant difference was between the sham-calcitonin group and the sham-vehicle group (laminae III–IV; p = 0.0163, laminae V–VI; p = 0.0163).

The results of the comparison between groups of c-Fos expression in laminae III–IV and laminae V–VI were similar to the results in laminae I–II, however, the only statistically significant difference was between the sham-calcitonin group and the sham-vehicle group. Significant differences in the numbers of c-Fos immunoreactive neurons in laminae III–IV and laminae V–VI were observed only between the sham-calcitonin group and the sham-vehicle group (laminae III–IV; p = 0.0163, laminae V–VI; p = 0.0163; Figure 7), indicating that the numbers of neurons that produced c-Fos after the formalin test in the sham-operated rats were decreased by calcitonin administration.

## Discussion

Subcutaneous injection of the rat hind paw with formalin is known to produce a biphasic response consisting of an early intense response (early phase) in the first five minutes, and a later moderate response (late phase) that is lasts from 20 to 60 min after the injection. The nociceptive response to formalin injection is matched by a corresponding biphasic increase in the activity of dorsal horn neurons after the injection [22]. The early phase is thought to be caused by stimulation of the peripheral nerve by the formalin, and the late phase is thought to be attributable to the secondary inflammation in the tissues [23]. C-fos mRNA was observed in the spinal cord after the formalin test, and expression peaked at 15–30 minutes in the superficial laminae of dorsal horn (laminae I–II) and at 30–60 minutes in the deep laminae (laminae V–VI). The expression of c-fos mRNA in those lesions appears to correspond to the early phase and the late phase of the response to the formalin test [24]. Altogether the appearance of c-Fos immunoreactive neurons in the superficial laminae of the dorsal horn shows the sensitization of post synaptic neurons after stimulation of peripheral nerve by formalin, indicating that direct transmission process of acute noxious stimulus. In the other hand, the appearance of c-Fos immunoreactive neurons in the deep laminae of the dorsal horn represents transmission of secondarily tissue inflammation.

In the present study the number of c-fos immunoreactive neurons in the dorsal horn of the spinal cord increased in response to the acute noxious stimulus in the OVX rats, particularly in the superficial lamina of the dorsal horn, indicating that OVX enhances nociception in rats. The mechanism of the hyperalgesia has not been well documented, although there have been some reports of hyperalgesia in OVX rat in behavioral studies [7,20,25]. A significant decline in estrogen hormone is known as a result of OVX [5]. Estrogen forms complexes with its receptors and controls expression of various genes by binding to specific sequences in the promoter region of the genes [26], and thus estrogen depletion affects the amount of gene products, such as peptides and receptors, associated with transmission of nociception. The number of serotonin receptors in the presynaptic area of dorsal horn has been reported to be decreased in OVX rats [27]. The report conjectures the fact with a disorder of the descending inhibitory serotonergic system that induces antinociception by inhibiting the release of neurotransmitters such as somatostatin [28]. Since c-Fos is a functional marker of nociception, our results indicate a change in that the transmission of noxious stimuli to secondary afferent neurons in OVX rats. Taken together, the results of this study suggest that the hyperalgesia in OVX rat is attributable to changes in the descending inhibitory serotonergic system.

The results of this present study also show that the repeated calcitonin injections have a suppressive effect that decreases the number of c-Fos producting neurons in the superficial laminae of the dorsal horn after the formalin test. Almost all experimental studies have examined only the acute anti-nociceptive effects of calcitonin [29-32], and administration consisted of a single centrally injections such as an intracerebroventricular injections [29,31], intrathecal injection [31,32] or injection into the periaqueductal gray matter [30]. These acute antinociceptive effects were thought as acting by those binding sites in central nervous system directry [33,34]. In clinical settings repeated peripheral injections for a month are required to treat the pain accompanying osteoporosis [9,35]. In behavioral studies, repeated systemic injections of calcitonin have been found to inhibit formalin-induced hypalgesia in rats, while single injections have had no effects [36] and repeated systemic injections of calcitonin have inhibited OVX-induced hypalgesia in rats [20]. In those studies calcitonin was effective when it injected repeatedly for about 4 weeks. These results are similar to the clinical effects of calcitonin reported in patients [9,35]. According to these facts, the duration and doses of calcitonin used in the present study were similar to those used clinically. In addition, it is reported in the experimental studies that the expression of c-Fos in the dorsal horn increased and the pain threshold decreased after formalin or yeast injection and skin incision, and administration of NSAIDs or anesthegia effects to improve pain threshold and inhibit c-Fos expression in the spinal cord [36-39]. Therefore, our results suggest that repeated systemic injection of calcitonin has an antinociceptive effect. However, the c-Fos expression is related with the reaction of the formalin test but dose not reflect the bone pain by postmenopausal osteoporosis in this study. There are studies that calcitonin has the effect of reducing bone pain associated with bone metabolic disorders, compression fracture and osteoporosis [1-4,40,41]. The bone pain induced by ovariectomy was not investigated in this study. In addition, the pain induced by the formalin test is quite different from the clinical situation and tissue damage is worse than the clinical situation. This is the limitation of this study, however, calcitonin was the effect of inhibiting formalin-induced c-Fos expression which is a strong stimulant. Thereby, the results in this study show the benefit of calcitonin treatment. There are studies that show calcitonin has an effect on patients with osteoarthritis (OA) in the clinical trail [42] and improves cartilage erosion in the experimental studies [43,44]. Elderly people have possibility of developing both osteoporosis and OA. Furthermore, calcitonin provides benefits of improving osteoporosis itself, bone pain, hyperalgesia induced by postmenopausal condition and OA. Calcitonin may provide the benefit of improving symptoms various ways. In addition, clinical trials for calcitonin treatment on the different conditions of each disease are needed.

It has been reported that the decrease in number of serotonin receptors in OVX rats recovered after repeated injection of calcitonin, and it was concluded that the reversal of the changes in serotonin receptors is one of the mechanisms of the analgesic actions of calcitonin [27]. Together with the results showing that calcitonin inhibits formalin-induced hypalgesia in a rat behavioral study [45], our results indicate that calcitonin has an antinociceptive effect in sham-operated rats. This result suggests that the antinociceptive mechanism of calcitonin is activation as well as restoration of the descending inhibitory serotonergic system.

The suppressive effect of calcitonin that caused the smaller number of c-Fos-producting neurons in the dorsal horn in our study was abolished by PCPA. The behavioral studies also demonstrated that the calcitonin-induced antinocciception was completely inhibited by intraperitoneal injection of PCPA [21,40]. Because PCPA is an inhibitor of serotonin biosynthesis, the amount of serotonin was decreased and the activity of the descending inhibitory serotonergic system was degraded after PCPA injection. Our results indicate that the involvement of central serotonergic system in the antinococeptive mechanism of calcitonin by immunohistochemical method, and is congruent with the report of antinociceptive mechanism through restoration of serotonin receptors [27].

The results in laminae III–IV and laminae V–VI were similar to the results in laminae I–II, however, the only statistically significant difference was observed between the sham-calcitonin group and the sham-vehicle group. Because the appearance of c-Fos in deep laminae (laminae V–VI) represents transmission of secondary tissue inflammation, our results suggest that repeated injection of calcitonin also has an antinociceptive effect on nociceptive stimulation by tissue inflammation. The fact that a significant difference observed only between the sham-calcitonin group and sham-vehicle groups may be result of the difference in antinociceptive effect of calcitonin between OVX rats and sham-operated rats and between restoration and activation of descending inhibitory serotonergic system. Further studies are necessary to clarify the antinococeptive effect of calcitonin.

## Conclusion

This is the first immunohistochemical study to investigate hyperalgesia in OVX rats and the antinociceptive effect of calcitonin. We concluded that the hyperalgesia in OVX rat is attributable to changes in the descending inhibitory serotonergic system, that repeated systemic administration of calcitonin has an antinociceptive effect, and that the descending serotonergic inhibitory system is involved in the mechanism of its antinociceptive action.

## Competing interests

The atuhors declare that they have no competing interests.

## Authors' contributions

All authors participated in the design of the study. BTK and MSE performed the studies and drafted the manuscript. MSE and SKO performed statistical analysis. SKO and SKI participated in coordination and helped to draft the manuscript. All authors have read and approved the final version of the manuscript.

## Pre-publication history

The pre-publication history for this paper can be accessed here:


